# Mediterranean Diet and COVID-19: Hypothesizing Potential Benefits in People With Diabetes

**DOI:** 10.3389/fendo.2020.574315

**Published:** 2020-09-16

**Authors:** Maria Ida Maiorino, Giuseppe Bellastella, Miriam Longo, Paola Caruso, Katherine Esposito

**Affiliations:** Department of Advanced Medical and Surgical Sciences, University of Campania “Luigi Vanvitelli”, Naples, Italy

**Keywords:** COVID-19, type 2 diabetes (T2D), mediterranean diet, inflammation, cytokines, glycemic control

## Introduction

The outbreak of the Coronavirus Disease 2019 (COVID-19) started in December 2019 in Wuhan (China) and has since spread in more than 200 countries. The pandemic was brought about by a novel virus causing severe acute respiratory syndrome coronavirus 2 (SARS-CoV-2). Among the comorbidities of people suffering from COVID-19, the most prevalent are diabetes, cardiovascular disease, and hypertension, all of which are associated with worse outcomes (1).

People with diabetes are at increased risk of severe viral respiratory tract infections, including the SARS-CoV, H1N1 influenza, and Middle East Respiratory Syndrome (MERS-CoV). The prevalence of diabetes in individuals with COVID-19 has been reported to range between nearly 10% and up to 30%, depending on the location of the study, population, age of participants in the studies, severity of illness, and method of testing (2). Moreover, diabetes has emerged as an important predictor of severity of the SARS-CoV-2, as the risk of fatal outcomes has been reported to be 50% higher in individuals with diabetes than in those without (3). Given the high transmission rate of SARS-CoV-2 and the global prevalence of diabetes, which affects nearly half a billion people worldwide, the coexistence of both COVID-19 and diabetes should be considered alarming, as it represents the combination of two pandemics.

## The Harmful Trio Linking COVID-19 and Diabetes: Impaired Immune Response, Inflammation, Pro-Thrombotic State

Both COVID-19 and diabetes are responsible for dysfunctional immune responses and generation of a pro-inflammatory and pro-thrombotic status which may lead to disease progression. People with COVID-19 generally present lymphopenia and increased neutrophil–lymphocyte ratio, consequent to the recruitment of immune cells from the blood and the infiltration of lymphocytes into the airways. This phenomenon is induced in the lungs by the release of inflammatory cytokines and chemokines [including Interleukin 6 (IL-6), interferon γ-inducible protein-10, macrophage inflammatory protein 1α (MIPα), MIP1β and monocyte chemoattractant protein-1] from the epithelial and endothelial cells and alveolar macrophage activated by the virus-linked pyroptosis. As a result, monocytes, macrophages and T cells are recruited to the site of infection, promoting further inflammation (together with the IFNγ produced by T cells) and establishing a pro-inflammatory feedback loop (4). It is also known that elevated levels of IL-6 and C-reactive protein (CRP), as well as increased coagulation activity, marked by high d-dimer concentrations, were also associated with varying degrees of severity of the disease.

Diabetes is thought to be a status of chronic inflammation induced by both hyperglycemia and insulin-resistance and characterized by high levels of pro-inflammatory cytokines, oxidative stress, and over-expression of adhesion molecules by endothelial cells (5, 6), which may predispose to an increased risk of infections. Poor glycemic control is also associated with an impaired immune response due to an inhibited lymphocytes ability to counteract different series of pathogenic stimuli and an alteration in monocyte/macrophage functions. Moreover, an imbalance between coagulation and fibrinolysis takes place in diabetes, with enhanced platelet aggregation and activation, favoring the development of a hypercoagulable pro-thrombotic state. Globally, all these alterations may represent the underlying conditions linking diabetes to other chronic conditions, including hypertension and cardiovascular diseases (5).

Ongoing clinical trials are currently evaluating the safety and efficacy of treatment strategies addressing the components of the harmful trio in the context of SARS-CoV-2; until now, however, no drug or vaccine has been officially approved for COVID-19 treatment. On the other hand, evidence from randomized controlled trials evaluating the effect of anti-inflammatory drugs on glycemic control or cardiovascular events in people with type 2 diabetes suggest only a modest effect on HbA1c reduction (5); whether patients with cardiovascular diseases and/or type 2 diabetes may have clinical benefit from marked reductions in circulating inflammatory markers remains also controversial. However, both the maintenance and intensification of the glyco-metabolic control in individuals with diabetes who have not been infected with the SARS-CoV-2 virus has suggested to be a means of primary prevention of COVID-19 disease (3).

## The Mediterranean Diet: A Healthy Dietary Pattern For People With Diabetes

Mediterranean diet, which represents the traditional eating habits of populations living around the Mediterranean Sea in the 1960s, including Greece and South Italy, has long been indicated as a dietary pattern able to preserve cardio-metabolic health. The Mediterranean diet is characterized by the high content in plant-based foods, olive oil as the main source of fat, low-to-moderate intake of fish, dairy products, and poultry, low consumption of red or processed meat, and low to moderate consumption of wine with meals (7). This dietary regimen is currently included among the eating patterns recommended by the American Diabetes Association for people with pre-diabetes or diabetes, showing favorable effects on HbA_1C_ reduction, weight, and lipids in a number of randomized controlled trials (RCTs). Moreover, the recent guidelines on diabetes, pre-diabetes, and cardiovascular diseases developed by the European Society of Cardiology in collaboration with the European Association for the Study of Diabetes state that a Mediterranean diet, rich in polyunsaturated and monounsaturated fats, should be considered to reduce cardiovascular events.

## Three Hypothetical Mechanisms Responsible For The Protective Effect of Mediterranean Diet Against COVID-19 In Diabetic People

### Mediterranean Diet Exerts Anti-inflammatory and Immunomodulatory Effects

Several epidemiological studies confirmed the anti-inflammatory and immunomodulatory effects of a Mediterranean pattern on several diseases associated with chronic low-grade inflammation. These counter the effects of several inflammatory markers and decrease the secretion of circulating and cellular biomarkers involved in the atherosclerotic process (8). A recent meta-analysis of 17 RCTs including 2,300 subjects reported a significant reduction in high-sensitive CRP [weight mean difference (WMD): −0.98 mg/l, 95% CI −1.48 to −0.49, *p* < 0.0001] IL-6 [WMD: −0.42 pg/ml, 95% CI −0.73 to −0.11, *p* = 0.008], and intracellular adhesion molecule-1 [WMD: −23.73 ng/ml, 95% CI −41.24 to −6.22, *p* = 0.008] in individuals assigned to Mediterranean diet, compared with those following a control intervention protocol (9). In two RCTs of people with obesity (10) and metabolic syndrome (11), the consumption of Mediterranean diet for 2 years was associated with a significant reduction of inflammatory markers, including CRP, IL-6, IL-7, and IL-18, as compared with a prudent diet. The MEditerranean DIet and Type 2 diAbetes (MÈDITA) study, provides further evidence of the anti-inflammatory and immunomodulatory effect of Mediterranean dietary pattern in the context of diabetes (12, 13). The MÈDITA trial was a long-term RCT conducted with a Mediterranean diet vs. a low-fat diet for primary prevention of antidiabetic drug need in 215 participants with newly-diagnosed type 2 diabetes. A significant 37% decrease in CPR, associated with a 43% raise in adiponectin was observed in the Mediterranean diet group after 1 year, while remaining unchanged in the low-fat diet group (12). In diabetic people with the highest scores of adherence to Mediterranean dietary pattern, as compared with the diabetic patients who showed the lowest adherence, the lower circulating CRP levels and higher circulating adiponectin concentrations suggested a “dose-dependent” anti-inflammatory effect (12). Moreover, the amelioration of the inflammatory milieu associated with Mediterranean diet was sustained over a 8.1 year-follow up, leading to an improvement of insulin resistance and glucose metabolism as compared with a low-fat diet (13).

### Mediterranean Diet Prevents Diabetes and Improves Glucose Control in Diabetic People

The most robust available evidence related to eating patterns for prevention of type 2 diabetes relies to Mediterranean diet, that, in 10 prospective studies, led to a 23% reduced risk of incident diabetes (14). Moreover, meta-analyses of RCTs reported that, compared with control diets, Mediterranean diets reduced HbA_1c_ levels by 0.30–0.47% in type 2 diabetes (7).

### Mediterranean Diet Reduces Cardiovascular Risk in Diabetes

In both interventional prospective studies and RCTs, Mediterranean diet has been associated with beneficial effects on weight circumference, blood lipids, and blood pressure. Moreover, in the PREDIMED study, involving people at high cardiovascular risk, of whom 49% had diabetes, a Mediterranean diet supplemented with olive oil or nuts led to a 28% reduction in the incidence of major cardiovascular events (15).

### Plausible Mechanisms

As both COVID-19 and diabetes are characterized by increased levels of pro-inflammatory cytokines, including CRP, IL-6 and TNF-α, treatment strategies effective in reducing inflammation may be pursued to prevent the risk of infection or blunt the severity of the disease in diabetic people. Among the components of Mediterranean diet, whole grains, vegetables and fruits, fish and “healthy” fats, including both monounsaturated (MUFA) and polyunsaturated (PUFA), are all associated with lower inflammation; on the other hand, processed red meat and dairy products, processed cheese, which are rich in saturated fatty acids and trans-fatty acids, may have pro-inflammatory properties (16).

The mechanisms by which Mediterranean diet produces its favorable effects in type 2 diabetes may depend mostly on the abundance of anti-inflammatory nutrients (PUFA, fiber, vitamins, minerals, antioxidants, and polyphenols), associated with the lower intake of pro-inflammatory nutrients (refined sugars and starches, trans fatty acids, high-density foods). Among PUFA, the omega-3 free fatty acids exert anti-inflammatory effects via oxygenated metabolites (oxylipins), which are specialized pro-resolving mediators (17). They include α-linolenic acid, derived from various plant sources, as well as eicosapentaenoic acid and docosahexaenoic acid, derived mainly from fish and seafood. Moreover, increased dietary fiber intake has been associated with reduced inflammatory markers (hs-CRP, IL-6, and TNF-α), and modifications in gut microbiota favoring health associated bacteria. There is also evidence supporting the protective role of vitamins against viral infection through different mechanisms (17). Furthermore, vitamin D facilitates the binding of the SARS-CoV-2 cell entry receptor ACE2 (angiotensin converting enzyme 2) to angiotensin II receptor type 1 (AGTR1), minimizing the number of viral particles that could attach to ACE2 and enter the cell, whereas vitamin C and vitamin E are well-known anti-oxidants agents, able to reduce the generation of reactive oxygen (ROS) and reactive nitrogen species (RNS) (17). Finally, polyphenols seem to interact with transcription factors, including NF-kB and Nrf-2, exerting anti-inflammatory and antioxidant effects, respectively (17).

The high content of Mediterranean diet in healthy food, combined with the low content of harmful food may be also responsible for the benefits on cardio-metabolic risk factors (i.e., blood pressure, lipids and lipoprotein, endothelial function, glucose levels) and cardiovascular events (7).

## Can Mediterranean Diet Protect Against Infections?

An adequate and balanced diet represents an important fundament for an optimal immune response. It helps maintain the antibody production and assure the assumption of nutrients able to modulate inflammatory and oxidative stress processes (17). Despite the proposed mechanisms seem to support the beneficial effects of Mediterranean diet against COVID-19 in people with diabetes, the relationship between this healthy dietary pattern and SARS-CoV-2 remains unexplored. Similarly, literary data linking Mediterranean diet to a reduced risk of infections are scanty. In the REasons for Geographic Differences in Stroke (REGARDS) cohort of 30,239 community-dwelling adults, of whom 20% had diabetes, a high Mediterranean diet-style adherence was associated with 26% lower risk of sepsis (18). Moreover, the only evidence of a favorable effects of Mediterranean diet on respiratory infections derives from a prospective study of 128 boys and girls aged 1–5 years included in a nutritional program aimed at “Learning to eat from the Mediterranean” (19). After 1 year, with the increased Kidmed index, which assessed the quality of the Mediterranean diet, the number of catarrhal episodes, the degree of intensity, the emergency and hospital admissions showed a positive and statistically significant evolution, suggesting that Mediterranean diet could improve outcomes of child with recurring colds and frequent inflammatory complications. On the other hand, several component of food associated with Mediterranean diet, including omega-3 PUFA, polyphenols, flavonoids and vitamin D, have demonstrated to reduce inflammation and improve immune response in both *in vitro* and *in vivo* studies of lung infections (20).

## Discussion

Whether the amelioration of the pro-inflammatory milieu associated with Mediterranean diet components may help to prevent or reduce the severity of infections, including COVID-19 diseases, remains unknown. Moreover, there are no studies confirming that, in diabetic people, the improvement of glycemic control associated with a high adherence to the Mediterranean dietary pattern may protect from lung infections or blunt the severity of the disease. However, there is evidence from a large retrospective study of individuals affected by COVID-19, including nearly 1,000 participants with type 2 diabetes, that well-controlled blood glucose was associated with lower mortality compared with individuals with poor glycemic control during hospitalization, supporting the correlation between improved glucose control and better outcomes in SARS-CoV-2 affected-patients with pre-existing diabetes (21). These findings need to be confirmed in prospective studies to validate the importance of a healthy dietary pattern, as Mediterranean diet is thought to be, as a valuable therapeutic strategy to improve the prognosis of diabetic people affected by COVID-19, at least in individuals who do not need to be treated in the context of intensive care units. Currently, the effective behavioral measures able to reduce the risk of COVID-19 remain physical distancing, adequate hygiene and use of mask.

Another crucial point refers to the adherence to the Mediterranean dietary pattern during the COVID-19 pandemic. The lockdown imposed by governments in many countries, including Italy, have profoundly changed the lifestyle habits and everyday behavior of people with and without diabetes. In consequence of the quarantine, the limited access to daily grocery shopping may have reduced the assumption of fruits, vegetables and fish, in favor of processed food. However, a recent survey conducted on 3,533 Italian individuals (age range 12–86 years) suggested that, during the lockdown, Italian people have paid attention to Mediterranean diet, maintaining a high nutritional quality especially in Northern and Central Italy, in which a lower BMI was reported compared with the areas of Southern Italy and the Islands (22). Moreover, subjects falling in the low, medium, and high adherence to the Mediterranean Diet, consumed more than 50% of olive oil, vegetables, and legumes, food highly representative of the Mediterranean dietary pattern, demonstrating that following a healthy diet is feasible even in emergency conditions. The choice of food associated with Mediterranean diet, rich in nutrients with antioxidant and anti-inflammatory activities, may be of paramount importance to reduce the susceptibility to develop viral infections (17).

Diabetes and the associated comorbidities are significant predictors of morbidity and mortality in patients with COVID-19. The prevention of diabetes together with the implementation of glyco-metabolic control in people with diabetes may represent useful strategies to prevent or blunt the severity of the infection in individuals affected by COVID-19. Thanks to its anti-inflammatory and immunomodulatory properties, Mediterranean diet provides a nutritional regimen potentially able to address the common soil of pathogenic factors linking diabetes to COVID-19, offering an opportunity to protect diabetic people from worse outcomes in the context of SARS-CoV-2. However, future studies are warranted to confirm the benefits of these dietary-linked effects on the risk of infections, including COVID-19.

## Author Contributions

MIM and GB drafted the manuscript. ML and PC contributed to drafting the manuscript and revised it for intellectual content. KE edited the manuscript and revised it for important intellectual content. All authors gave the final approval of the version submitted and agreed to be accountable for all aspects of the work in ensuring that questions related to the accuracy or integrity of any part of the work they have done.

## Conflict of Interest

The authors declare that the research was conducted in the absence of any commercial or financial relationships that could be construed as a potential conflict of interest.
